# Hyaluronic acid stimulation of stem cells for cardiac repair: a cell-free strategy for myocardial infarct

**DOI:** 10.1186/s12951-024-02410-x

**Published:** 2024-04-04

**Authors:** Seon-Yeong Jeong, Bong-Woo Park, Jimin Kim, Seulki Lee, Haedeun You, Joohyun Lee, Susie Lee, Jae-Hyun Park, Jinju Kim, Woosup Sim, Kiwon Ban, Joonghoon Park, Hun-Jun Park, Soo Kim

**Affiliations:** 1 Brexogen Research Center, Brexogen Inc., Songpa‑gu, Seoul, 05855 South Korea; 2 Department of Biomedicine & Health Sciences, The Catholic University of Korea, 222 Banpo-daero, Seoho-gu, Seoul, 06591 Republic of Korea; 3 Catholic High-Performance Cell Therapy Center and Department of Medical Life Science, College of Medicine, The Catholic University of Korea, 222 Banpo-daero, Seoho-gu, Seoul, 06591 Republic of Korea; 4 Department of Biomedical Science, City University of Hong Kong, Kowloon Tong, Hong Kong; 5 Graduate School of International Agricultural Technology, Institutes of Green-Bio Science and Technology, Seoul National University, Pyeongchang, Gangwon-do 25354 South Korea; 6 Division of Cardiology, Department of Internal Medicine, The Catholic University of Korea, 222 Banpo-daero, Seocho-gu, Seoul, 06591 Republic of Korea

**Keywords:** Extracellular vesicles, Induced Mesenchymal stem cells, Myocardial infarction

## Abstract

**Background:**

Myocardial infarction (MI), a representative form of ischemic heart disease, remains a huge burden worldwide. This study aimed to explore whether extracellular vesicles (EVs) secreted from hyaluronic acid (HA)-primed induced mesenchymal stem cells (HA-iMSC-EVs) could enhance the cardiac repair after MI.

**Results:**

HA-iMSC-EVs showed typical characteristics for EVs such as morphology, size, and marker proteins expression. Compared with iMSC-EVs, HA-iMSC-EVs showed enhanced tube formation and survival against oxidative stress in endothelial cells, while reduced reactive oxygen species (ROS) generation in cardiomyocytes. In THP-1 macrophages, both types of EVs markedly reduced the expression of pro-inflammatory signaling players, whereas HA-iMSC-EVs were more potent in augmenting anti-inflammatory markers. A significant decrease of inflammasome proteins was observed in HA-iMSC-EV-treated THP-1. Further, phospho-SMAD2 as well as fibrosis markers in TGF-β1-stimulated cardiomyocytes were reduced in HA-iMSC-EVs treatment. Proteomic data showed that HA-iMSC-EVs were enriched with multiple pathways including immunity, extracellular matrix organization, angiogenesis, and cell cycle. The localization of HA-iMSC-EVs in myocardium was confirmed after delivery by either intravenous or intramyocardial route, with the latter increased intensity. Echocardiography revealed that intramyocardial HA-iMSC-EVs injections improved cardiac function and reduced adverse cardiac remodeling and necrotic size in MI heart. Histologically, MI hearts receiving HA-iMSC-EVs had increased capillary density and viable myocardium, while showed reduced fibrosis.

**Conclusions:**

Our results suggest that HA-iMSC-EVs improve cardiac function by augmenting vessel growth, while reducing ROS generation, inflammation, and fibrosis in MI heart.

**Graphical Abstract:**

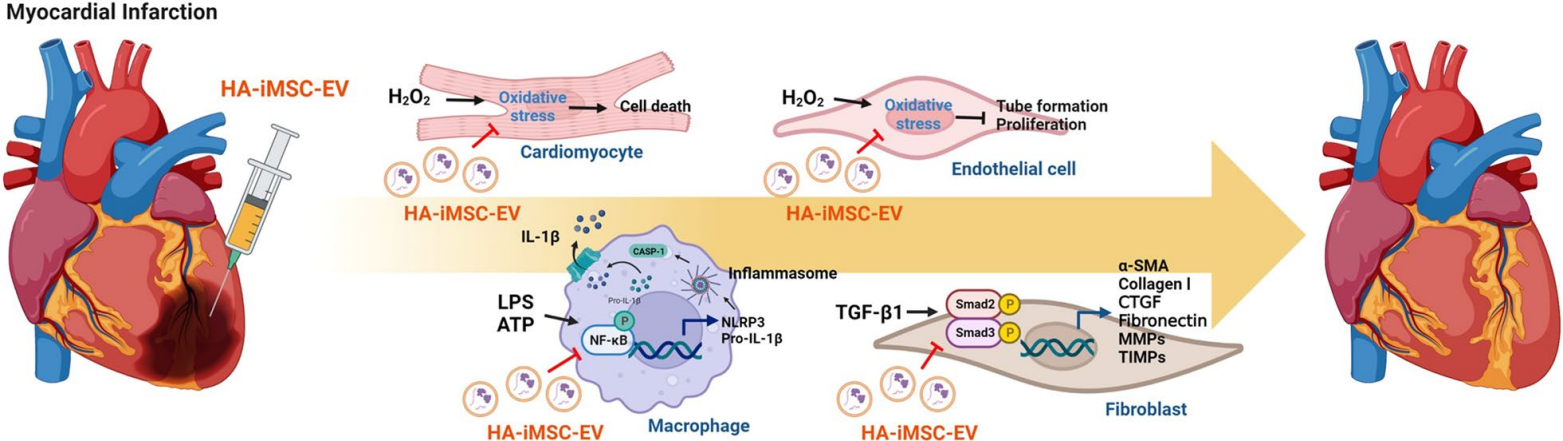

**Supplementary Information:**

The online version contains supplementary material available at 10.1186/s12951-024-02410-x.

## Introduction

Ischemic heart disease (IHD) is a leading cause of death worldwide [1]. MI leads to myocardium loss, pathological remodeling, dysfunctional cardiac function, and heart failure [2]. MI is manifested by myocyte loss, which triggers a cascade of immune-inflammatory pathways and cellular processes such as complement activation, the generation of ROS, and the activation of inflammasomes. The recovery begins with the action of inflammatory cells, which replace the necrotic myocardium with granulation tissue. Also, fibroblasts generate a new collagen matrix, which eventually results in the formation of the post-infarction scar in the infarcted area [3–5].

Recent studies have demonstrated that mesenchymal stem cells (MSCs) can reduce inflammation, fibrotic, and injury to the parenchyma, thus contributing to tissue repair [6–12]. Earlier clinical studies have been shown that transplanted stem cells can promote tissue regeneration via engraftment in the myocardium, not via differentiation into cardiomyocytes [13, 14]. Despite the benefits, MSCs have not translated well into clinical practice because clinical trials have consistently failed to produce conclusive outcomes possibly due to the difficulties in preparing large scale of homogenous cells that can be applicable for therapeutical purposes [15]. In this regard, MSC-like cells derived from pluripotent stem cells (iMSCs) have tremendous advantages owing to their homogeneity and stable growth profile [16, 17]. These properties make it possible to generate a large quantity of clonally derived iMSCs in a scalable manner [18]. Several animal studies using iMSCs have shown significant benefits in tissue regeneration and repair [19–22].

There is Increasing evidence that the therapeutic effect of MSCs is exerted by their EVs, which are recognized as key mediator for cell-to-cell interaction [23]. EVs hold several potential advantages over cell therapies, such as smaller size, lower complexity, lack of nuclei, increased stability, easier production, and longer preservation [24–26]. Also, the risk of direct administration of cell therapy product such as oncogenesis and thromboembolism can be reduced by using EVs [25]. Furthermore, the profile of cargo biomolecules of EVs can be altered by preconditioning the original cells [27], which makes the EVs’ therapeutic outcome can be optimized (Additional file 1: Figs S1–S3).

The cardiac extracellular matrix (ECM) supports the heart mechanically and regulates molecular signals that control spatial and temporal dynamics [28, 29]. Among various families, HA is an essential component of the cardiac ECM that promotes the repair of injured tissues [30]. Therapeutically, HA-hydrogels have been implicated in advancing cardiac repair processes by promoting angiogenesis, reducing inflammation, and supporting transplanted cells [30–32].

In this study, we aimed to investigate whether EVs from hyaluronic acid-primed iMSCs (HA-iMSC-EVs) can alleviate in rat model of MI. We found that HA-iMSC-EVs reduced apoptosis, blocked M1 macrophage polarization, inhibited fibrotic changes, improved cardiac function, enhanced interstitial capillary growth, and reduced aberrant tissue remodeling in MI heart. We also conducted in vitro studies using relevant cells.

## Methods

### Generation of HA-iMSCs and HA-iMSC-EVs

iMSC were prepared as described in our previous study [17]. Briefly, iMSC was differentiated from iPSC that was established from WJ-MSC transduced with Yamanaka factors using CytoTune iPS 2.0 Sendai Reprogramming kit (Thermo Fisher Scientific, Waltham, MA, USA). Established iMSCs were cultured in high-glucose Dulbecco’s Modified Eagle’s Medium (DMEM; HyClone, Chicago, IL, USA) supplemented with 15% Fetal Bovine Serum (FBS; HyClone) and 1% antibiotic–antimycotic solution (Thermo Fisher Scientific) at 37 °C in 5% CO_2_ and 95% humidified air. Upon reaching 90% confluence, the cells were detached using TrypLE Express (Thermo Fisher Scientific) and seeded at a density of 10,000 cells/cm^2^. Next day, the cells were treated with 40 μg/mL Hyaluronic acid (Sigma aldrich, St Louis, MO, USA) for 24 h, after which the media were aspirated, and the cells were washed with Dulbecco’s Phosphate Buffered Saline (DPBS; HyClone). The culture media were replaced with phenol red-free DMEM (Gibco, Waltham, MA, USA) supplemented with 15% EV-depleted FBS. EV-depleted FBS was prepared as described previously [33]. After 3 days of incubation, the culture medium was harvested, and HA-iMSC-EVs were isolated by ultracentrifugation as described previously [34].

### Flow cytometry

To confirm whether HA preconditioned iMSCs express the typical cell surface markers for MSCs, HA-iMSCs were trypsinized and washed twice prior to resuspension in PBS containing 2% FBS. Cells were adjusted to 1 × 10^6^ in 100 µL of cell suspension. For cell surface labeling, cell suspensions were incubated at 4 °C for 30 min with antibodies (dilution, 1:20). APC conjugated mouse anti-human CD73, PE-conjugated mouse anti-human CD105, FITC-conjugated mouse anti-human CD45, PE-conjugated mouse anti-human CD31, and APC conjugated mouse anti-human CD34 antibodies were supplied by eBioscience (Waltham, MA, USA) and, APC-Cy7-conjugated mouse anti-human CD90 was supplied by BioLegend (San Diego, CA, USA). To characterize surface proteins of EVs, HA-iMSC-EVs analysis was performed using human MACSPlex Exosome Kit (Miltenyi Biotec, Bergisch Gladbach, Germany) according to the manufacturer’s instructions. Flow cytometric analysis was conducted using an Attune NxT flow cytometer (Thermo Fisher Scientific).

### Cryo-transmission electron microscopy

To confirm the morphology and size of EVs, Cryo-transmission electron microscopy (TEM) was performed as described in our previous study [34]. Briefly, the HA-iMSC-EVs suspension was placed on a grid and blotted and were visualized at 36,000 × magnification using a Talos L120C FEI transmission electron microscope (Thermo Fisher Scientific) at 120 kV.

### Nanoparticle tracking analysis

To measure the particle size distribution and concentration of EVs, Nanoparticle Tracking Analysis (NTA) was performed using Zetaview^®^BASIC NTA-Nanoparticle Tracking (Particle Metrix, Inning am Ammersee, Germany). For the analysis, EVs were serial diluted in sterile PBS to reach the optimal volume for NTA. The standard control was set as Sensitivity: 80, Frame Rate: 30, Shutter: 100, Temperature: 23 °C.

### Labeling of HA-iMSC-EVs with DiR and DiD and fluorescent imaging

HA-iMSC-EVs were incubated with 1 μg/mL DiR or DiD buffer according to the protocol provided by Lipophilic Tracers (Invitrogen, Waltham, MA, USA) as described previously [34]. 500 μg of DiR-labeled HA-iMSC-EVs were resuspended in 0.05 mL of phosphate buffered saline (PBS) and intravenously or intramyocardially injected into rats. DiR-labeled HA-iMSC-EVs were detected at 6 and 24 h using an In Vivo Imaging System (IVIS, Perkin Elmer, Waltham, MA, USA). Human umbilical venous endothelial cells (HUVECs) or neonatal rat cardiac fibroblasts (NRCFs) were treated with DiD-labeled HA-iMSC-EVs. After 24 h, DiD-labeled HA-iMSC-EVs were observed under a Nikon Eclipse Ti2-U fluorescence microscope (Nikon, Tokyo, Japan).

### Cell viability and tube formation

HUVECs (3 × 10^3^ in 96 well plates) were treated with 500 μM of H_2_O_2_ for 2 h. Culture medium were then replaced with serum-free EGM2 Endothelial Growth Medium (LONZA, Basel, Switzerland) containing 100 μg/mL of iMSC-EVs or HA-iMSC-EVs, and cultured for 48 h at 37 °C and 5% CO_2_ incubator. Cell Counting Kit-8 (CCK-8; Enzo Life Sciences, Farmingdale, NY, USA) analysis was performed according to the manufacturer’s instructions. For tube-forming assay, the HUVECs were resuspended in a serum-free EGM2 medium and seed in 96 well plates (1 × 10^4^ cells/well) pre-coated with Matrigel (Corning, NY, USA). Tube formation was confirmed after 16 h. 

### Cytotoxicity and reactive oxygen species assay

Cardiomyocytes derived from iPSCs (iPSC-CMs) were treated with 500 μM H_2_O_2_ for 2 h, and subsequently incubated with iMSC-EVs or HA-iMSC-EVs in serum-free DMEM for 24 h. Next, the CellROX® Reagent (Thermo Fisher Scientific) was mixed with serum-free DMEM to a final concentration of 5 μM and added to the culture for 30 min. Following staining, the cells were fixed in 4% paraformaldehyde (Fujifilm Wako Chemicals, Richmond, VA, USA) for 10 min and washed three times with DPBS. Nuclei and cell bodies were counterstained with NucBlue^™^ Fixed Cell stain or CellTracker^™^ (Invitrogen), respectively. After this process, all samples were observed using a Nikon Eclipse Ti2-U (Nikon, Tokyo, Japan), and the percentage of ROS-positive cells was analyzed based on nuclear intensity.

### Real time PCR

Total RNA was isolated from the M1 or M2 polarized THP-1 macrophages treated with HA-iMSC-EVs 100 µg/mL for 24 h using TRIzol^®^ (Ambion, Waltham, MA, USA). M1 macrophages were induced using 100 ng/mL of lipopolysaccharides (LPS) and 20 ng/mL of interferon-gamma (IFN-γ) after 200 ng/mL of phorbol 12-myristate 13-acete (PMA) treatment of THP-1 cells, and M2 macrophages were induced using 20 ng/mL of interleukin 4 (IL-4) and 20 ng/mL of interleukin 13 (IL-13) after 200 ng/mL of PMA treatment. GAPDH was used as a reference to normalize the differences in mRNA quantity in each sample. The relative gene expression levels were analyzed using the comparative 2^−ΔΔCt^ method. hGAPDH: F; 5′-GTCGGAGTCAACGGATTTGG-3, R; 5′-AGTTGAGGTCAATGAAGGGGTC-3, hTNF-α: F; 5′-GAGCTGAACAATAGGCTGTTCCCA-3, R; 5′-AGAGGCTCAGCAATGAGTGACAGT-3, hCXCL10: F; 5’-TGGCATTCAAGGAGTACCTCTC-3, R; 5′-TGATGGCCTTCGATTCTGGA-3, hTGF-β: F; 5′-CCCAGCATCTGCAAAGCTC-3, R; 5′-GTCAATGTACAGCTGCCGCA-3, IL-10: F; 5′-TGAAAACAAGAGCAAGGCCG-3, R; 5′-GCCACCCTGATGTCTCAGTT-3, hCCL22: F; 5′-GCGTGGTGTTGCTAACCTTCA-3, R; 5′-GGGGAGCAGCTATAATGGCA-3.

### Western blot

HA-iMSCs and HA-iMSC-EVs were lysed in NP40 or RIPA lysis buffer (Thermo Fisher Scientific) supplemented with protease inhibitors (Thermo Fisher Scientific). Protein concentration was measured using the Bradford Assay Reagent (Thermo Fisher Scientific) according to the manufacturer’s protocol. Samples were diluted using 4 × Laemmli buffer (Bio-Rad Laboratories, Hercules, CA, USA) and heated at 100 °C for 10 min. Proteins were loaded and separated on precast polyacrylamide Mini-PROTEAN TGX gels (Bio-Rad Laboratories) and transferred to PVDF membranes (Bio-Rad Laboratories). The membranes were blocked with EveryBlot Blocking Buffer (Bio-Rad Laboratories) for 5 min and then treated overnight with primary antibodies at 4 °C. All primary antibodies were diluted in the EveryBlot Blocking Buffer. Antibodies against GM130, Histone H3 (Cell Signaling Technology, Leiden, The Netherlands), CD63, CD9, calnexin, Cytochrome C (Abcam, Cambridge, UK), TSG101, and CD81 (Invitrogen, Waltham, MA, USA) were used as the primary antibodies. Western blotting for all target proteins, except CD81 was performed under reducing conditions. The membranes were washed for 10 min for five times and then treated with the secondary antibodies for 1 h. After the membranes were washed for 10 min for five times, the target proteins were detected using the ECL Select Western Blotting Detection Reagent (GE Healthcare, Little Chalfont, UK) and analyzed using the ChemiDoc Imaging System (Bio-Rad Laboratories). For detecting inflammasome proteins in THP-1 treated with LPS 1 μg/mL, adenosine triphosphate (ATP) 5 mM and HA-iMSC-EVs 400 μg/mL, primary antibodies against NLRP3 (NACHT, LRR, and PYD domain-containing protein 3, also known as cryopyrin), pro-caspase-1, interleukin-1 beta (IL-1β), p65, GAPDH (Abcam, Cambridge, UK), caspase-1 (Novus biologicals, Centennial, CO, USA), alpha-tubulin and phospho-p65 (Cell signaling, Leiden, The Netherlands) were used. The fibrosis-related proteins were detected in NRCF-treated with transforming growth factor-beta (TGF-beta) 1 μg/mL (for 48 h) (Sigma Aldrich, St Louis, MO, USA) and 400 μg/mL HA-iMSC-EVs (for 24 h). Collagen 1, Fibronectin, TIMP-1 (Invitrogen), MMP-2 (Abcam), phospho-SMAD2, and SMAD2/3 (Cell Signaling) were used as primary antibodies. Western blotting of all target proteins, except collagen 1, was performed under reducing conditions.

### Bioinformatics

Metascape pathway and process enrichment analysis (http://metascape.org) were used to investigate the potential biological functions and mechanisms of EV proteins. The Reactome gene set was used as an ontology source, and enriched terms with a p-value < 0.01, minimum count of three, and enrichment factor > 1.5 were collected and grouped into clusters. In addition, a Connectivity Map (https://clue.io) was employed to analyze HA-iMSC-EV-specific proteins and identify approved drugs that were similar to HA-iMSC-EVs. Based on this analysis, the potential indications for EVs were predicted.

### Myocardial ischemia–reperfusion injury and HA-iMSC-EVs treatment

All animal experiments were approved by the Institutional Animal Care and Use Committee (IACUC) of The Catholic University of Korea (approval number: CUMC-2020–0063-03). To produce an ischemia–reperfusion (I/R) model, male Fischer 344 rats (8-weeks old, 160–180 g, Koatech, South Korea) were anesthetized with 2% inhaled isoflurane and intubated via the trachea using an 18-gauge intravenous catheter. The proximal portion of the left anterior descending artery was surgically occluded for 1 h through ligation with a 7–0 PROLENE suture, followed by coronary reperfusion through the release of the tie. 50 μL of HA-iMSC-EVs were injected into the myocardium at a dose of 10 mg/kg or 20 mg/kg, and 500 μL of HA-iMSC-EVs were intravenously injected at a dose of 20 mg/kg, 5 min before releasing the tie. In the group that received two injections, the material was delivered to the myocardium using the same method 7 days after the first injection.

### Measurement of myocardial infarct size

2,3,5-Triphenyltetrazolium chloride (TTC) and Evan’s Blue staining was performed to assess the early cardioprotective effects of HA-iMSC-EVs. Rats were anesthetized and mechanically ventilated according to previously described methods. After 60 min of ischemia and 24 h of reperfusion, the suture thread around the LAD artery, previously used in surgery, was retired, and Evan’s blue dye (9% in PBS) was administered intravenously. After 15 min, the heart was excised and immediately incubated for 10 min at—4 °C. The groups were divided into two according to the timing of the injection: (1) under ischemic conditions, the EVs were injected 5 min before reperfusion, and (2) under reperfusion conditions, the EVs were injected 5 min after reperfusion. And each group was divided into two groups according to the route of the injection: intramyocardial (IM) or intravenously (IV) injection. The heart was sliced into 3 sections, each approximately 2 mm in thickness, and incubated with 2% TTC for 30 min in a dark, humid environment at 37 °C. Following three washes, tissues were fixed in 4% paraformaldehyde. Evan’s blue staining indicated non-infarcted myocardium, which was stained deep blue, whereas the viable myocardium was stained red by TTC. The necrotic myocardium appeared white on TTC staining. The ImageJ software was used to quantify the area at risk (AAR) and necrotic areas.

### Echocardiography

The I/R-induced cardiac injury was functionally evaluated using echocardiography. Rats were anesthetized with 2% isoflurane, and data were recorded using a transthoracic echocardiography system equipped with a 15 MHz L15-7io linear transducer (Philips, Amsterdam, Netherlands, Affiniti 50G). Serial echocardiograms were performed at baseline and 4 h, 1, 2, 3 and 5 weeks after surgery. The experiment was conducted in a blinded manner, with the echocardiography operator unaware of the group allocation. Left ventricular systolic function was assessed by calculating the ejection fraction (EF) and fractional shortening (FS) using the following formulas: EF (%) = [(LVEDV-LVESV)/LVEDV] × 100 and FS (%) = [(LVEDD-LVESD)/LVEDD] × 100.

### Hemodynamic measurements

Hemodynamic measurements were conducted at the 5-week endpoint prior to euthanasia. The rats were anesthetized using 2% isoflurane, and thoracotomy was performed without causing bleeding. A 26-gauge needle was used to puncture the left ventricular (LV) apex, and a 2F conductance catheter (Millar, Houston, TX, USA, SPR-838) was inserted into the LV. Continuous recording of the pressure–volume (PV) parameters was accomplished using a PV conductance system (Emka TECHNOLOGIES, Paris, France, MPVS Ultra) connected to a digital converter (ADInstruments, Dunedin, New Zealand, PowerLab 16/35). Load-independent cardiac function measurements were obtained with different preloads induced by inferior vena cava occlusion using a needle holder. To assess the parallel conductance after hemodynamic measurements, hypertonic saline (50 µL of 20% NaCl) was injected into the left jugular vein. Blood was collected from the LV into a heparinized syringe and placed into cuvettes for conversion of the conductance signal to volume using a catheter. Calibration of the parallel and cuvette conductance was used to confirm the absolute volume of the rat.

### Immunohistochemical staining

The hearts were fixed with 4% paraformaldehyde overnight, and paraffin blocks were prepared. Cross-sections of the heart, measuring 4 µm, were made using a microtome (Leica, Wetzlar, Germany, LRM2255), starting from the top of the apex. Immunofluorescence was performed to evaluate the capillary density of the injured hearts. After deparaffinization and rehydration, antigen retrieval was performed using a target retrieval solution in a decloaking chamber. The sections were then incubated with a diluted primary antibody at 4 °C overnight. The primary antibodies used were mouse anti-cardiac troponin T (1:200) (Abcam, Cambridge, UK; ab8295) and goat anti-CD31 (1:200) (R&D Systems, Minneapolis, MN, USA; AF3628). After washing the samples four times with phosphate-buffered saline (PBS), the secondary antibody was added and incubated for 90 min at room temperature in the dark. The secondary antibodies used in this study were donkey anti-mouse IgG (H + L) highly cross-adsorbed secondary antibody, Alexa Fluor 488 (1:500) (Invitrogen, Waltham, MA, USA, A-21202), and rabbit anti-goat IgG (H + L) cross-adsorbed secondary antibody, Alexa Fluor 594 (1:500) (Invitrogen, A-11080). Following another wash with PBS, the sections were stained with an anti-fade mounting medium containing 40,6-diamidino-2-phenylindole (DAPI) (Vector Laboratories, Burlingame, CA, USA, H-1200–10) for nuclear staining and then mounted onto slides.

### Masson’s trichrome staining

To determine the fibrotic area, wall thickness scar area, and viable myocardium of the injured hearts, Masson's trichrome staining (Sigma-Aldrich, Saint Louis, MO, USA, HT15) was carried out. Paraffin slides were preincubated overnight at 37 °C before deparaffinization and rehydration. After deparaffinization and rehydration, the paraffin sections were fixed again for 1 h and 30 min in Bouin's solution at 56 °C, followed by washing with tap water for 20 min. The sections were then stained with Weigert's iron hematoxylin solution for 15 min at room temperature, followed by staining with Biebrich scarlet acid fuchsin solution for 20 min at room temperature. Finally, sections were counterstained with aniline blue for 15 min and incubated in 1% acetic acid for 2 min at room temperature. The collagen fibers appeared blue, and the viable myocardium appeared red. Heart section imaging was conducted using a slide scanner (3DHISTECH Ltd., Budapest, Hungary, PANNORAMIC MIDI II), and all areas, including the fibrotic area, were quantified using ImageJ software.

### Statistical analyses

In vitro statistical analyses were performed by one-way analysis of variance (ANOVA) using GraphPad Prism Software and data were expressed as means ± standard deviation (SD). Alternatively, one-way analysis of variance (ANOVA) performed using SPSS version 18.0 (IBM Corp., Armonk, NY, USA) and the data were expressed as means ± standard error (SE). A value of p < 0.05 were considered statistically significant. In vivo data are presented as the mean ± standard error of the mean (SEM). Statistical significance (p < 0.05) was determined by one-way ANOVA and unpaired t-test using GraphPad Prism Software.

## Results

### Characterization of HA-iMSCs and HA-iMSC-EVs

**Fig. 1:**
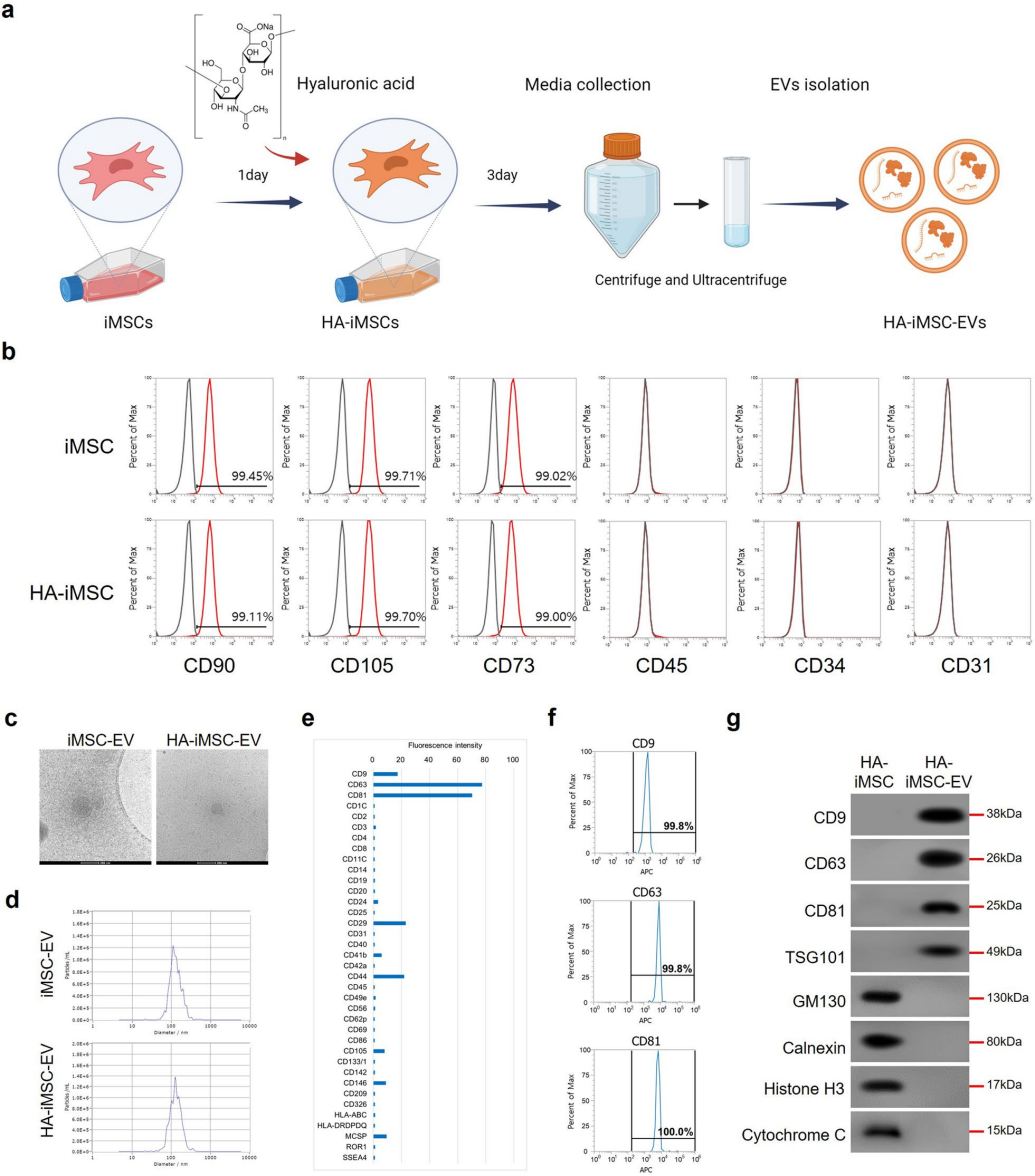
Characterization of HA-iMSCs and HA-iMSC-EVs. **a** Scheme of the HA-iMSC-EVs manufacturing. HA-iMSC-EVs were isolated from the hyaluronic acid primed iMSC culture medium using ultracentrifugation. **b** Flow cytometric examination of markers positive (CD90, CD73, and CD105) or negative (CD45, CD31, and CD34) for iMSCs and hyaluronic acid preconditioned iMSCs (HA-iMSCs). **c** Representative image of iMSC-EV and HA-iMSC-EV observed using cryo-TEM. Scale bar = 200 nm. **d** NTA result of iMSC-EVs and HA-iMSC-EVs showed the average size is 138 nm. **e**, **f** Flow cytometric analysis of surface marker profile of HA-iMSC-EVs (**e**), and representative marker of EVs: CD9, CD63 and CD81 (**f**). **g** Immunoblot analysis of HA-iMSCs and HA-iMSC-EVs for markers of extracellular vesicles (CD9, CD63, CD81, and TSG101) or cellular organelles (GM130, calnexin, Histone H3, and Cytochrome C)

In this study, we isolated EVs from iMSC to overcome the limitations of MSCs in the same way as described in previous publications [17, 34]. Additionally, to enhance the therapeutic potential of EVs, we used HA as a priming factor for the preconditioned media (Fig. 1a) [30, 35–37]. After HA priming, we conducted flow cytometric analysis to confirm changes in the expression of typical MSC markers. The results showed that HA-primed iMSCs had typical MSC, such as CD90, CD73, and CD105, and were negative for CD45, CD34, and CD31 (Fig. 1b). We also characterized EVs derived from HA-iMSC (HA-iMSC-EVs), which showed that HA-iMSC-EVs had typical EV characteristics, including a spheroid shape, 50–200 nm size, and were positive for tetraspanin proteins, including CD9, CD63, and CD81 (Fig. 1c–g).

### Pro-angiogenic effect of HA-iMSC-EVs on endothelial cells

**Fig. 2:**
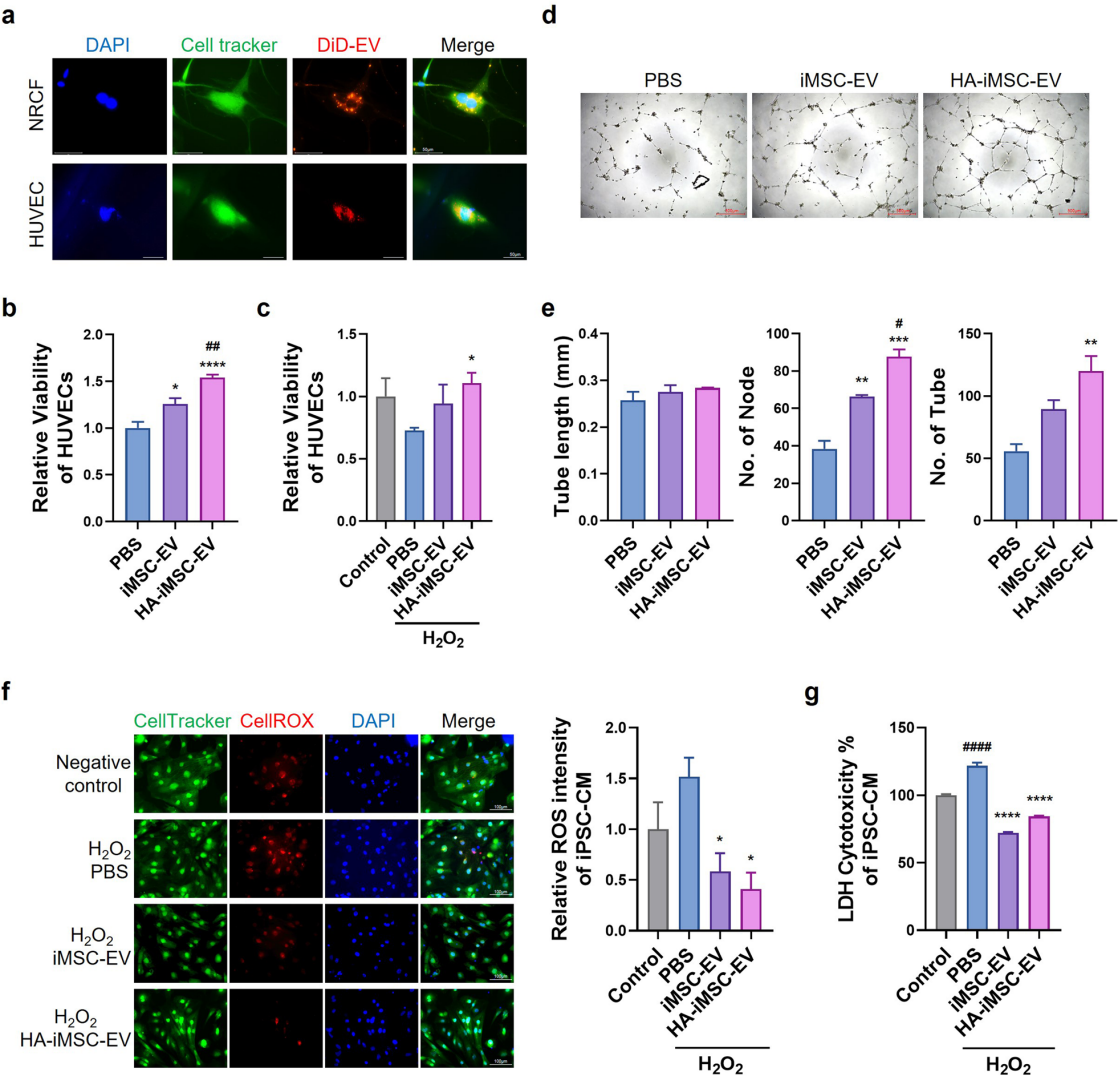
Multifunctional effects of HA-iMSC-EVs on the various cell types. **a** Incorporation of DiD-labeled HA-iMSC-EVs into NRCF and HUVEC. The uptake of DiD-labeled HA-iMSC-EVs was examined after 24 h treatment with 100 μg/mL EVs (600 × magnification). **b**–**c** Enhanced effect of HA-iMSC-EVs on endothelial cell proliferation. Comparison of the viability of HUVEC (**b**) and H_2_O_2_-damaged HUVEC (**c**) after treatment with PBS, iMSC-EVs, or HA-iMSC-EVs. Data are presented as mean ± SD (n = 6 and n = 3, respectively). *p < 0.05; ****p < 0.0001 *vs* PBS; ^##^ p < 0.01 *vs* iMSC-EV; one-way ANOVA. **d**–**e** Tube formation by HUVEC treated with iMSC-EVs or HA-iMSC-EVs. **d** Representative images of HUVEC tube formation. **e** Quantification of tube length (left), number of nodes (middle), and number of tubes (right). Data are presented as the mean ± SD (n = 3). **p < 0.01; ***p < 0.001 *vs* PBS; # p < 0.05 *vs* iMSC-EV; ns, not significant; one-way ANOVA. **f**–**g** Cytotoxicity reducing effects of HA-iMSC-EVs on hiPSC-CMs. **f** Representative image and fluorescence intensity quantification graph of ROS levels in hiPSC-CMs with H_2_O_2_-induced oxidative damage after treatment with PBS, iMSC-EVs, or HA-iMSC-EVs. *p < 0.05 *vs* PBS. **g** Comparison of LDH levels representing cytotoxicity in H_2_O_2_-injured hiPSC-CMs after treatment with PBS, iMSC-EVs, or HA-iMSC-EVs. Data are presented as the mean ± SD (n = 3). ****p < 0.0001 *vs* PBS; #### p < 0.0001 *vs* Control; one-way ANOVA

First, to confirm whether HA-iMSC-EVs could be incorporated into cells, NRCFs and HUVECs were treated with DiD-labeled EVs for 24 h. The DiD-labeled EVs were detected by red fluorescence in the cytoplasm (Fig. 2a). Next, to check whether the penetrated EVs could affect cell proliferation, HUVECs were treated with EVs for 48 h, and then viable cells were checked by cell counting kit-8 analysis. The relative viable cell levels increased with EVs’ treatment, and the viable cell levels in cells treated with HA-iMSC-EVs were higher than those treated with iMSC-EVs (Fig. 2b). This means that cells treated with HA-iMSC-EVs proliferated more than those treated with iMSC-EVs. In addition, HA-iMSC-EVs treatment increased the number of relatively viable HUVECs under oxidative stress conditions (Fig. 2c). To confirm the effects of the EVs on angiogenesis, we performed tube formation assays using HUVECs. HA-iMSC-EVs also enhanced the tube formation capacity of iMSC-EVs by increasing the number of nodes and tubes (Fig. 2d, e). These results suggest that HA-iMSC-EVs promote the angiogenesis and proliferation of HUVECs.

### Cytotoxicity-reducing effect of HA-iMSC-EVs on cardiomyocytes

To evaluate the cardioprotective effects under oxidative stress, we measured the levels of ROS and lactate dehydrogenase (LDH) levels in iPSC-CMs after the treatment of iMSC-EVs or HA-iMSC-EVs. iPSC-CMs were used after confirming the surface phenotype of cardiomyocyte (CM) including cardiac troponin T (cTnT) and-actinin (sFig. 1a). CellROX dye intensity was measured to quantify the ROS level, which showed that red dye intensity decreased in iMSC-EV- or HA-iMSC-EV-treated iPSC-CMs (Fig. 2f). LDH levels, indicating cytotoxicity, also decreased in iPSC-CMs treated with iMSC-EVs or HA-iMSC-EVs (Fig. 2g). Additionally, when HA-iMSC-EVs were treated with H_2_O_2_ damaged primary neonatal rat cardiomyocytes (NRCMs), the relative viability of the NRCMs increased 48 h after EVs’ treatment (Fig. 2). These data indicate that iMSC-EVs and HA-iMSC-EVs have a protective effect against oxidative stress in CMs. Taken together, these data indicated that HA-iMSC-EVs have multifunctional abilities to support cardiac protection and regeneration by demonstrating the effects in promoting proliferation and angiogenesis and reducing cytotoxicity.

### Anti-inflammatory effects of HA-iMSC-EVs on macrophages

**Fig. 3:**
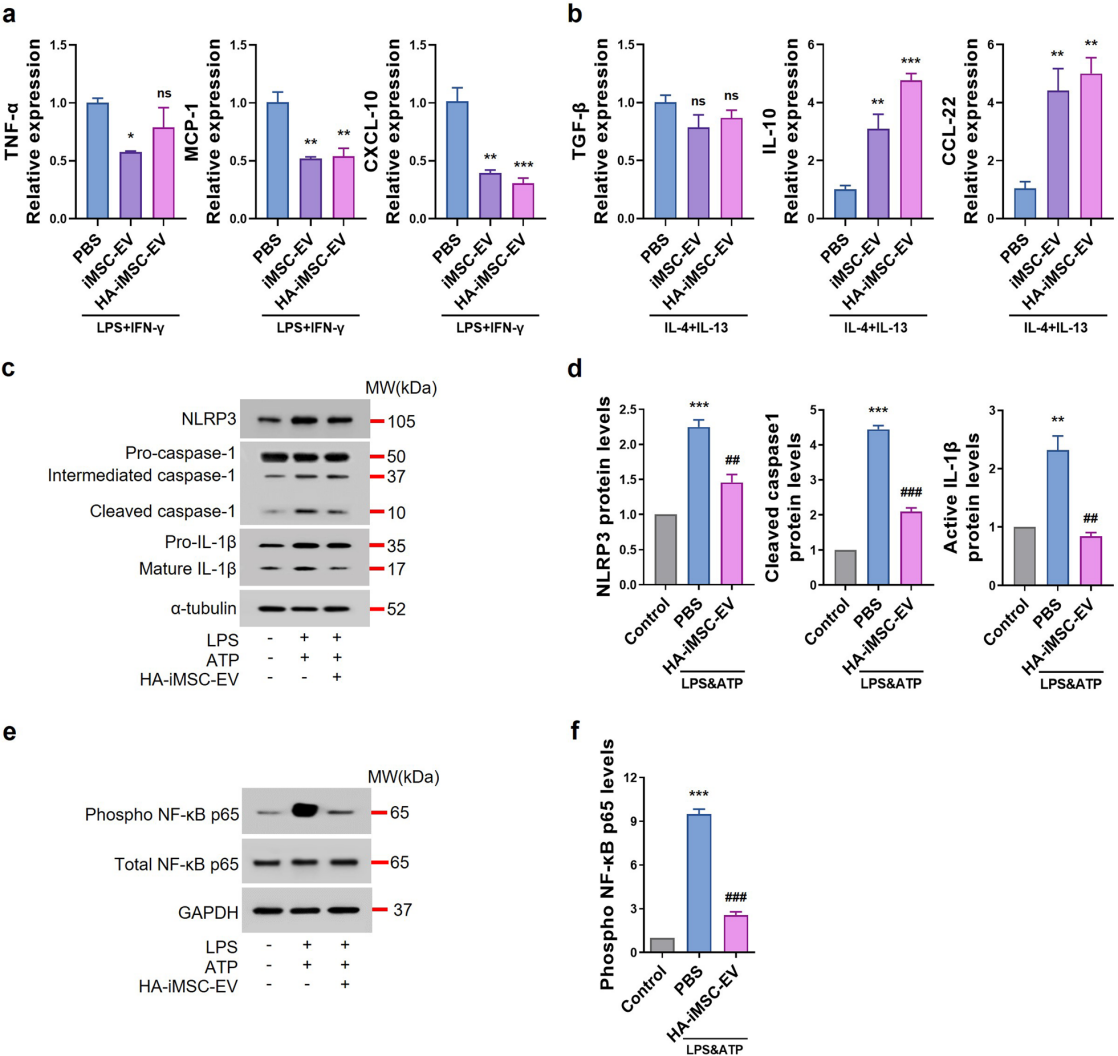
Anti-inflammatory effect of HA-iMSC-EVs on macrophages. **a**, **b** Effects of HA-iMSC-EVs on macrophage polarization of THP-1 cells treated with LPS and IFN-γ (**a**) or IL-4 and IL-13 (**b**). **a** Comparison of the relative mRNA expression levels of M1 polarization-related genes (TNF-α, MCP-1, and CXCL-10). **b** Comparison of relative mRNA expression levels of M2 polarization-related genes (TGF-β, IL-10, and CCL-22). Data are presented as the mean ± SD (n = 3). *p < 0.05; **p < 0.01; ***p < 0.001; ns, not significant; one-way ANOVA. **c**–**f** Inflammasome-related protein expression in THP-1 cells treated with LPS, ATP, and HA-iMSC-EVs. **c**, **d** Western blot images represent that the inflammasome sensor protein NLRP3 and effector proteins caspase-1 and IL-1b were reduced by 400 μg/mL of HA-iMSC-EVs treatment in THP-1 activated by 1 ug/mL of LPS and 5 mM of APT (**c**) and the graphs show the quantitation results (**d**). Mean ± SE, n = 3, ***p < 0.001; **p < 0.01 *vs* Control, ^###^p < 0.001; ^##^p < 0.01 *vs* PBS; one-way ANOVA. **e**, **f** Suppression of p65 phosphorylation in THP-1 macrophages treated with LPS and ATP using HA-iMSC-EVs. The protein levels of phosphorylated p65 decreased in THP-1 cells treated with HA-iMSC-EVs (**e**), and the graph shows the quantitation results (**f**). Mean ± SE, n = 4, ***p < 0.001 *vs* Control, ^###^p < 0.001 *vs* PBS; one-way ANOVA

Next, we confirmed the effects of EVs on inflammation by assessing macrophage polarization in THP-1 cells after EVs’ treatment. To confirm the effects of EVs on M1 macrophage polarization, THP-1 cells pretreated with PMA for 2 d were treated with LPS and IFN-γ together with EVs. After 24 h, the mRNA expression levels of M1 macrophage-related genes, such as TNF-α [38], MCP-1 [39], and CXCL-10 [40] were reduced in the HA-iMSC-EVs group, similar to those in the iMSC-EV group (Fig. 3a). To confirm the effects on M2 macrophage polarization, THP-1 cells treated with PMA for 2d were treated with IL-4 and IL-13 together with EVs. After 24 h, the mRNA expression levels of the M2 macrophages related genes such as IL-10 [41] and CCL22 [42] were increased in the HA-iMSC-EVs group, but not TGF-β, which was known to induce fibrosis [43] and M2 polarization [44] (Fig. 3b). These results suggest that iMSC-EVs and HA-iMSC-EVs suppress inflammation by modulating M1/M2 macrophage polarization.

Macrophages infiltrate the damaged area after MI, and the inflammasome in M1 macrophages promotes cardiac remodeling [45]. To evaluate the anti-inflammatory effects of HA-iMSC-EVs by inhibiting inflammasomes in THP-1 cells, we performed western blotting to measure changes in inflammasome-associated protein levels after HA-iMSC-EVs treatment of THP-1 cells treated with LPS and ATP. The levels of the inflammasome sensor protein NLRP3, effector proteins caspase-1, and IL-1β were decreased by HA-iMSC-EVs treatment (Fig. 3c, d), and phosphorylated NF-κB p65 levels were also decreased (Fig. 3e, f). These data suggested that HA-iMSC-EVs suppress inflammation via inflammasome downregulation.

### Anti-fibrotic effects of HA-iMSC-EVs on fibroblast

**Fig. 4:**
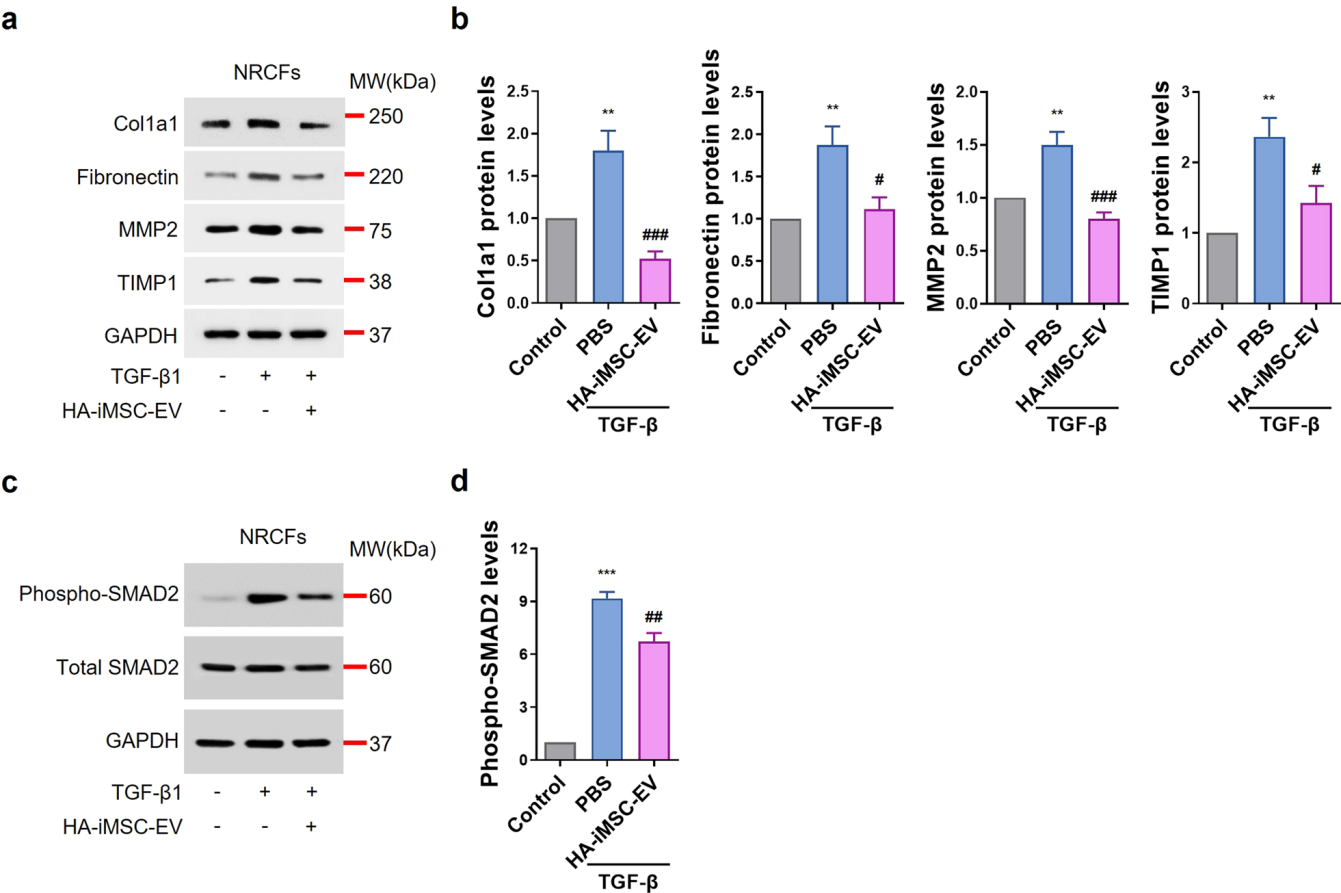
Anti-fibrotic effects of HA-iMSC-EVs on NRCF undergoing fibrosis. **a**, **b** Western blot image show the expression of fibrosis-related proteins such as Collagen 1, Fibronectin, MMP2, and TIMP1 were suppressed by 400 μg/mL of HA-iMSC-EVs treatment in NRCF treated with 1 μg/mL of TGF-β1 (**a**) and the graphs show quantification results (**b**). Mean ± SE, n = 4, **p < 0.01 *vs* Control, ^###^p < 0.001; ^#^p < 0.05 *vs* PBS; one-way ANOVA. **c**, **d** Western blot image shows that the expression of phosphorylated SMAD2 was suppressed in NRCF treated with TGF-β1 by HA-iMSC-EVs, and the graph shows the quantification results. Mean ± SE, n = 4, ***p < 0.001 *vs* Control, ^##^p < 0.01 *vs* PBS; one-way ANOVA

When the heart is damaged after MI, signals such as danger-associated molecular patterns (DAMP) or ROS increase, and immune cells respond to them, causing inflammation and fibrosis by inflammatory cytokines [46]. Remodeling proceeds through these processes, and the heart loses its function [46]. In our previous study, we confirmed that TGF-β expression did not change in M2 macrophages treated with HA-iMSC-EVs (Fig. 3b). To confirm whether HA-iMSC-EVs had an anti-fibrotic effect, we measured changes in the expression of fibrosis-related proteins in NRCFs treated with TGF-β and HA-iMSC-EVs. Proteins induced by TGF-β, including Collagen 1, Fibronectin, MMP2, and TIMP1 were reduced by HA-iMSC-EVs treatment for 24 h (Fig. 4a, b). SMAD2 phosphorylation was also decreased by HA-iMSC-EVs treatment (Fig. 4c, d). These results suggest that HA-iMSC-EVs inhibit the fibrotic change of cardiomyocytes induced by TGF-β1.

### Assessment of drug potential of HA-iMSC-EVs

**Fig. 5:**
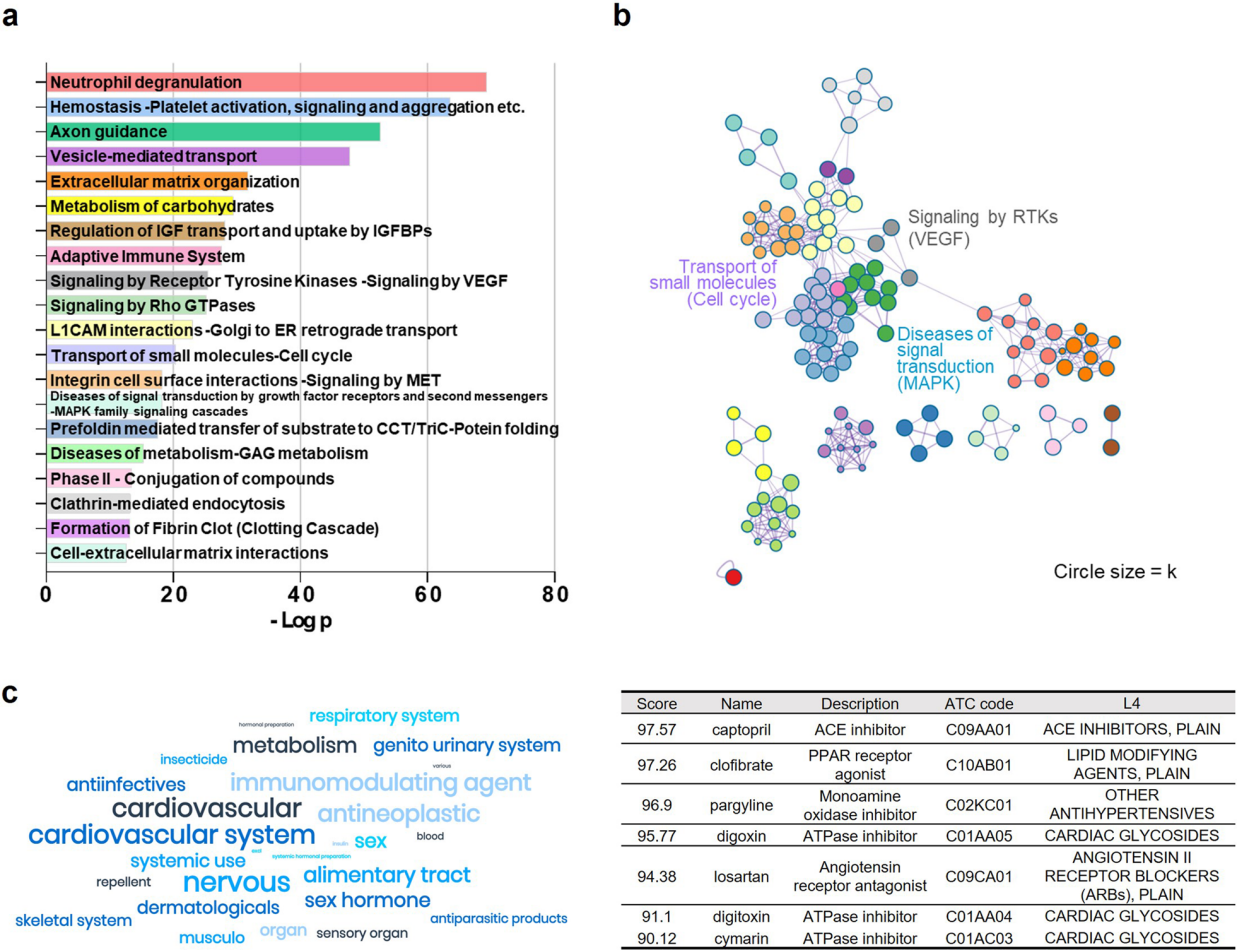
Proteomic analysis and functional enrichment of HA-iMSC-EV proteins. **a** Top 20 enriched pathways and process terms of HA-iMSC-EVs. The bar graph represents -log_10_(p-value). **b** Clustering analysis of angiogenesis, cell cycle, and MAPK signal transduction. Pathways were clustered at a minimum overlap of 3, p = 0.01, and a minimum enrichment of 1.5. The colors of the nodes matched the colors of each pathway, as shown in (**a**). **c** Connectivity map analysis of HA-iMSC-EV proteins. The potential indications for HA-iMSC-EVs classified according to the ATP level 1 classification were implemented as a word cloud (left). The cardiovascular drugs with the highest connectivity scores to HA-iMSC-EVs are listed in Table (right)

The potential biological functions and mechanisms of HA-iMSC-EVs were analyzed using EV proteins. HA-iMSC-EV proteins were qualitatively and quantitatively identified using LC–MS/MS, and functional enrichment analysis was conducted using Metascape [47]. In total, 796 EV proteins were identified, with 244 being HA-iMSC-EV-specific proteins. The pathway and process enrichment analysis revealed significant clustering of vesicle-mediated transport related to EV biogenesis (p = 1.59 × 10^–48^), signaling by vascular endothelial growth factor (VEGF) for angiogenesis (p = 3.15 × 10^–26^), and cell proliferation-related cell cycle (p = 4.19 × 10^–21^) and MAPK family signaling cascades (p = 5.45 × 10^–19^) (Fig. 5a, b). Using the Anatomical Therapeutic Chemical (ATC) classification, we identified drugs that produced biological responses similar to those induced by HA-iMSC-EVs in the nervous and cardiovascular systems (Fig. 5c). Specifically, we identified 76 drugs in the nervous system and 66 drugs in the cardiovascular system, with the highest frequencies. Notably, Captopril had the highest connectivity score at 97.57, followed by pargyline at 96.9, digoxin at 95.77, losartan at 94.38, and cymarin at 90.12. These drugs are classified as ACE or ATPase inhibitors at an ATC level of 4. These findings suggest that HA-iMSC-EVs have therapeutic potential for cardiovascular diseases.

### Cardiac protection effect of HA-iMSC-EVs against myocardial ischemia–reperfusion injury

**Fig. 6:**
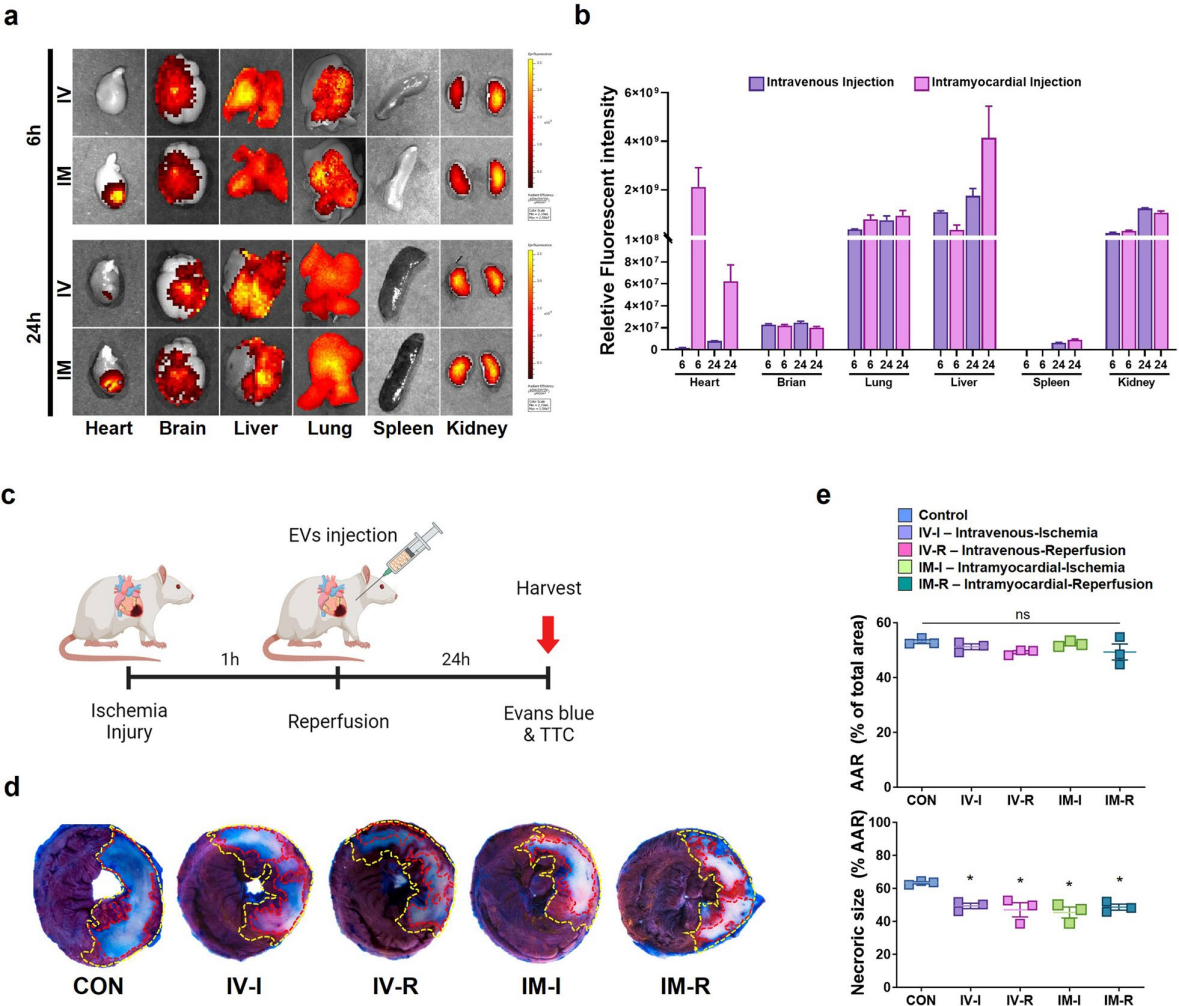
Cardiac protection effect of HA-iMSC-EVs against myocardial ischemia–reperfusion injury. **a**, **b** In vivo tracking of HA-iMSC-EVs. The localization of fluorescently labeled HA-iMSC-EVs was visualized after 6 or 24 h of intravenous (IV, upper) and intramyocardial (IM, lower) administration (**a**). **b** Bar graph shows the relative fluorescent intensity. **c** Experimental Process. HA-iMSC-EVs (20 mg/kg) were injected intravenously or intramyocardially 5 min before or after reperfusion. **d** Representative images of TTC/Evan’s blue staining 24 h after reperfusion. **e** The area at risk (AAR) was not significantly different between the I/R control and HA-iMSC-EVs injection groups, but necrotic size (white) was significantly reduced in the HA-iMSC-EVs injection group. * p < 0.05, compared to the PBS treatment group by unpaired t-test. Error bars show means ± SEM. n = three animals per group. ns, not significant

First, we investigated the degree of retention of HA-iMSC-EVs in the myocardium after direct injection into the myocardium (IM) and intravenous (IV) injection. When comparing the relative fluorescence intensity in the heart, brain, liver, lungs, pancreas, and kidneys, we confirmed that the intensity of HA-iMSC-EVs at 6 h after injection was approximately 40 times higher in the hearts of the IM injection group than in the IV injection group (Fig. 6a, b). After 24 h after injection, there was no difference in the intensity according to the injection route in the brain, lungs, pancreas, and kidneys, but the IM injection group showed more than a 7.9-fold increase of intensity in the hears compared with the IV injection group. To verify the initial myocardial protective effect of HA-iMSC-EVs after I/R injury, they were injected via each route 5 min before or after reperfusion (Fig. 6c). In tissues harvested 24 h after injection, there were no differences in the therapeutic effects between the HA-iMSC-EVs treatment groups depending on the injection route and timing, but the HA-iMSC-EVs-treated animals show significantly reduced necrotic size compared to the control group (Fig. 6d, e). These findings suggest that the HA-iMSC-EVs treatment protect against tissue necrosis in I/R-injured hearts.

### HA-iMSC-EVs restores impaired cardiac function

**Fig. 7:**
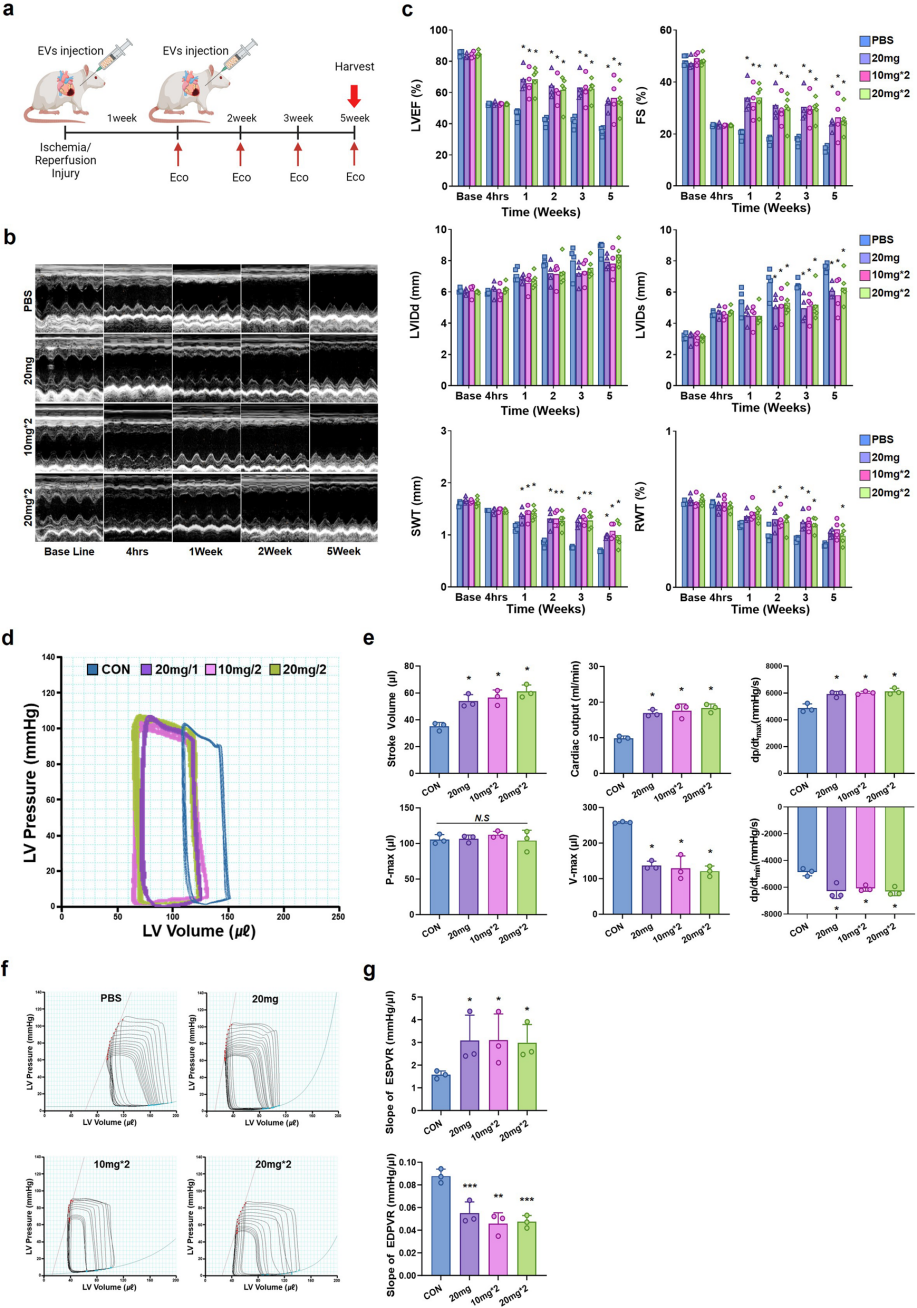
Injection of HA-iMSC-EVs improved cardiac function and reduced adverse cardiac remodeling. **a** Experimental Process. HA-iMSC-EVs (10 mg/kg or 20 mg/kg) were injected intramyocardially 5 min before reperfusion. The second injection was administered 1 week after the first treatment. **b** Representative M-mode echocardiography images at baseline (4 h) and 1, 2, and 5 weeks after EVs injection. **c** Differences in left ventricular ejection fraction (EF), fractional shortening (FS), septal wall thickness (SWT), end-diastolic (LVIDd), and end-systolic dimensions (LVIDs) between baseline and 5 weeks after EVs injection. * p < 0.05, compared with the PBS treatment group by one-way ANOVA. Error bars show means ± SEM. n = 4–5 animals per group. **d** Representative pressure–volume loops obtained from catheter-based left ventricular P–V measurements five weeks after IR injury. **e** Cardiac output, stroke volume, volume max, pressure max, and systolic and diastolic functions as measured by the maximal and minimal rates of pressure change during isovolumic relaxation (dP/dtmax and dP/dtmin). **f** Measurement of cardiac contractibility through inferior vena cava occlusion. **g** Slope of end-systolic pressure–volume relationship (ESPVR) and end-diastolic pressure–volume relationship (EDPVR). * p < 0.05; **p < 0.01; ***p < 0.001, compared with the PBS treatment group by Bartlett test in R. Error bars show means ± SEM. n = three animals per group

Considering the way to increase the effect of HA-iMSC-EVs treatment, which has an initial protective effect in I/R model, we decided to deliver HA-iMSC-EVs intramyocardially twice at 7 day intervals and performed echocardiography to track cardiac function. The regimen of HA-iMSC-EVs treatment was as follows: single 20 mg/kg, 10 mg/kg twice, or 20 mg/kg twice. After baseline echocardiographic examination, I/R modeling was induced, and cardiac function was evaluated at 4 h, 1, 2, 3, and 5 weeks (Fig. 7a). The cardiac function did not show significant differences between the groups in the echocardiographic results 4 h after I/R modeling. However, after 5 weeks of follow-up, all three HA-iMSC-EVs groups showed significantly improved left ventricular ejection fraction (LVEF) and fractional shortening (FS), as well as significantly decreased left ventricular internal diastolic dimension (LVIDd) and left ventricular internal systolic dimension (LVIDs) compared to the PBS control group. Septal wall thickness (SWT) was also improved compared to that in the PBS control group (Fig. 7b, c).

Next, we evaluated cardiac contractility via PV loop analysis, which allows for the independent measurement of LV hemodynamic pressure and volume during systole and diastole by directly inserting a catheter into the LV. Five weeks after I/R injury, treatment with HA-iMSC-EVs improved cardiac function and protected against adverse cardiac remodeling compared to the PBS control group (Fig. 7d, e). The HA-iMSC-EVs group exhibited significantly increased hemodynamic parameters, including cardiac output and stroke volume, during diastole, indicating improved cardiac function. Additionally, the group showed a lower maximum diastolic volume (V-max), a cardiac remodeling index representing blood volume in the LV at maximum diastole, indicating the inhibition of adverse cardiac remodeling. There was no significant difference in maximum pressure (P-max) in the LV at maximum systole across all groups. Cardiac function measures, such as dP/dtmax and dP/dtmin, were significantly improved in the HA-iMSC-EVs groups compared to the PBS group. Moreover, the inferior vena cava occlusion test revealed that the HA-iMSC-EVs group had a steeper slope for the end-systolic pressure–volume relationship (ESPVR) and a gentler slope for the end-diastolic pressure–volume relationship (EDPVR), as depicted in (Fig. 7f, g), suggesting enhanced load-independent heart contractility and ventricular compliance due to HA-iMSC-EVs treatment.

### HA-iMSC-EVs improve left ventricular remodeling and increase blood vessel density

**Fig. 8:**
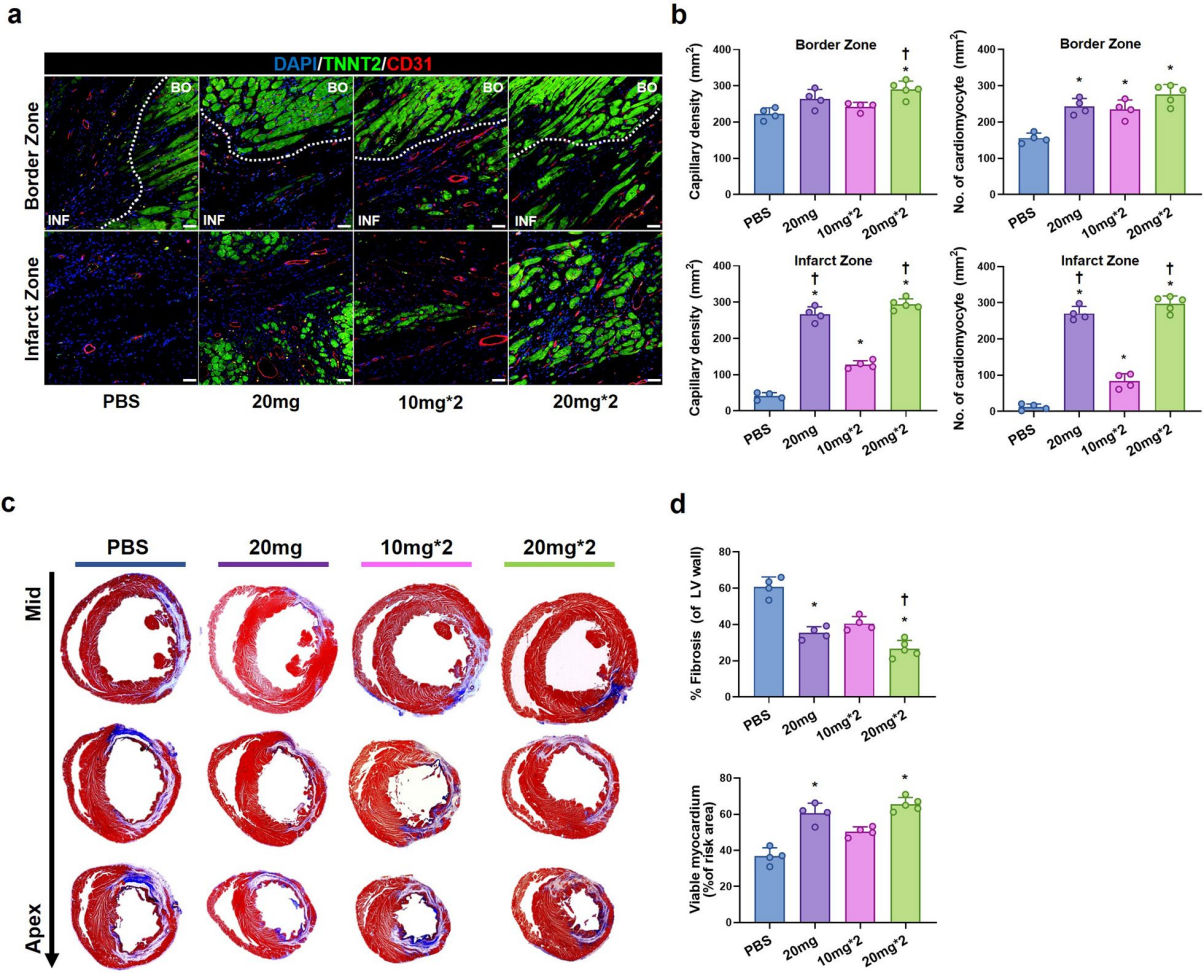
HA-iMSC-EVs increases blood vessel density and improves left ventricular remodeling after I/R injury. **a**, **b** Immunohistochemical staining for Cardiac Troponin T (TNNT2, green) and CD31 (red) in the border zone (upper) and infarct zone (lower) (**a**) and quantification of capillary density and a number of cardiomyocytes (**b**). **c**, **d** Representative images from the four experimental groups showing cardiac fibrosis after staining with Masson’s trichrome in the hearts harvested 5 weeks after I/R injury (purple and blue, scar tissue; red, viable myocardium, **c**) and quantification of the viable myocardium area and fibrosis area (**d**). n = 4–5 animals per group. * p < 0.05, compared to the PBS treatment group; † p < 0.05, compared to the 2 times 10 mg injection group by one-way ANOVA. Error bars show means ± SEM. n = 4–5 animals per group

To investigate whether HA-iMSC-EVs could increase blood vessel density and protect the myocardium in I/R-injured hearts, we performed immunostaining for CD31 and cardiac troponin T to visualize capillaries and cardiomyocytes, respectively. The results demonstrated that the treatment of HA-iMSC-EVs provide a significant increase of capillary density within the infarct zone compared to the control group, which was dependent on the dosage and the number of administration (Fig. 8a, b). However, in the border zone, the difference in capillary density was only significant in the HA-iMSC-EVs 20 mg/kg twice injection group compared to other groups. Cardiomyocyte densities in both border and infarct zone were also significantly increased in the HA-iMSC-EVs treatment groups compared to that in the PBS control group. Notably, in the infarct zone, the treatment of HA-iMSC-EVs provided a significant increase of viable cardiomyocytes in the same manner of the change of capillary densities (Fig. 8a, b). At 5 weeks, Masson’s trichrome staining revealed an increased viable myocardial area, a smaller area of fibrosis, and a thicker infarcted wall in the HA-iMSC-EVs groups (Fig. 8c, d). Taken together, these data indicating that the administration of the HA-iMSC-EVs improves the cardiac function with prohibiting tissue damage in the rat I/R model.

## Discussion

The purpose of this study was to explore whether HA-iMSC-EVs can promote the cardiac repair after MI, and to elucidate their possible mechanisms underlying their therapeutic outcome. We report that HA-iMSC-EVs met minimal guideline for EVs [48]. In endothelial cells, HA-iMSC-EVs showed increased ability to support tube formation as well as recovery from oxidative stress-induced cytotoxicity as compared with iMSC-EVs. The latter effects were also demonstrated in cardiomyocytes. HA-iMSC-EVs reduced inflammation in THP-1 macrophages, and also decreased SMAD2 phosphorylation and the expression of fibrosis markers in cardiomyocytes undergoing fibrotic change. Fluorescence tracking showed that HA-iMSC-EVs was delivered to myocardium after intravenous or intracardial injection, with the latter increased intensity. In vivo study showed that intramyocardial injections of HA-iMSC-EVs enhanced cardiac function while attenuating adverse cardiac remodeling. Detailed tissue study showed that HA-iMSC-EVs led to an increased capillary vessel formation and viable myocardium, while showed reduced fibrosis in infarcted hearts.

The protocols for generating autologous iPSCs and its subsequent differentiation into iMSCs in a scalable manner have not been standardized [49]. First, selecting the optimal cell type for iPSC generation can be a critical issue because the differentiation potential of iPSCs can be affected by the origin of the donor cell [50]. In this study, we used Wharton’s jelly (WJ)-derived MSCs for iPSC generation, after which were then reprogrammed into iPSCs. WJ-MSCs have several advantages over MSCs from other tissues in various aspects. WJ-MSCs are derived from the umbilical cord; therefore, abundant WJ-MSCs are readily available. In addition, the umbilical cord is discarded after birth, which provides an ideal source of stem cells that can be collected in a noninvasive, ethical, and painless manner [51, 52]. Moreover, it was demonstrated that the expression of pluripotency markers was higher than that in other sources, indicating that they are more developmentally primitive [53, 54]. In addition, WJ-MSCs are easier to isolate and expand in vitro than MSCs derived from other sources. After establishment, iPSCs were differentiated into iMSCs by removing pluripotency factors, resulting in the generation of iMSCs. iMSCs exhibit plastic adherence, express MSC surface markers, and can differentiate into osteoblasts, adipocytes, and chondroblasts [49, 55], which satisfy the minimal criteria for human MSCs proposed by the International Society of Cellular Therapy [56]. Previous studies, including ours, have shown that iMSCs can be maintained for extended periods without losing their doubling capacity, whereas primary MSC stop proliferating after several passages [55]. Additionally, iMSCs have a single-cell origin, which provides suitable conditions for producing homogeneous, quality-controlled EVs [57]. Thus, iMSCs are more advantageous than MSCs for producing EVs therapeutics.

One strategy to bolster the therapeutic potential of MSCs is their priming or preconditioning in vitro through exposure to hypoxia and heat shock, cytokines, and growth factors, pharmacological or chemical agents, biomaterials, or different culture conditions before transplantation into the hearts [35, 58]. The underlying mechanism of the priming effects is that MSCs exposed to these applications have short-term memory and remember a priming stimulus, even after moving to different environments, and change their phenotypic directions that are therapeutically beneficial [59]. In this regard, modulation of the biochemical and biophysical microenvironments may influence the fate of MSCs and enhance their therapeutic potential [58]. The invention of biomaterials that may guide MSC fate towards the desired phenotype and offer a microenvironment that allows for structural and biochemical cellular support is highly correlated with MSC use in tissue engineering, which improves tissue repair [58, 60]. Reproducible, biocompatible, and clinically applicable biomaterials for cell culture should be able to treat specific diseases.

Obviously, the molecular contents of EVs can be changed by priming of parental cells [61]. For example, it was shown that EVs from hypoxic cells exerted enhanced cardioprotective functions by suppressing the expression of pro-apoptotic genes p53 and BAK1, thus inhibiting CM apoptosis [62]. EVs obtained from miR-146a-modified adipose-derived stem cells (ADSC) have been shown to attenuate myocardial damage by suppressing the local inflammatory response through inhibition of the release of proinflammatory cytokines (IL-6, IL-1, and TNF-α). In addition, ADSC-EVs improve cardiac function by arresting CM apoptosis via the downregulation of early growth response factor 1 [63]. Huang et al. have discovered that EVs from atorvastatin-pretreated MSC (MSC-ATV-EVs) ameliorated cardiac dysfunction and reduced infarct area by diminishing IL-6 and TNF-α levels, promoting angiogenesis, and preventing apoptosis following MI. MSC-ATV-EVs are enriched with lncRNA H19, which regulates miR-675 expression and activation of pro-angiogenic factors [64]. Thus, HA-iMSC-EVs are biologically suitable for targeting cardiovascular diseases.

Bioinformatics analysis in the present study showed that HA-iMSC-EVs are enriched with proteins involved in pathways such as ECM organization, VEGF signaling, cell cycle regulation, and MAPK, all of which are essential for vascular and tissue survival [65–67]. Importantly, the connectivity map [68, 69] suggested that HA-iMSC-EVs share pharmacological functions with several drugs associated with cardiovascular diseases/development. HA is an unsulfated glycosaminoglycan with excellent viscoelasticity, high moisture retention capacity, biocompatibility, and hygroscopic properties [70]. Most cells in the body synthesize HA at some point in their cell cycle, implicating it in several fundamental biological processes. Wang et al. reported that HA oligosaccharides improve angiogenesis by upregulating VEGF secretion and myocardial function reconstruction after MI through the polarization of M2-type macrophages [71]. Le et al. also reported that HA-based microrods provide local biochemical and biomechanical signals to reprogram fibroblasts and attenuate cardiac fibrosis [72]. Recent study also reported that Hapln1-expressing epicardial cells were responsible for the processing and organization of HA within the ECM, which has recently been implicated in heart regeneration [73]. Furthermore, HA priming increased the trafficking, adhesion, and internalization of MSC EV into injured target cells, enhancing the therapeutic potency of the EV. HA may act as a bridge between MSC EV and target cells, allowing the EV to be internalized [37]. Together with these previous findings and the results from the present study, HA-iMSC-EVs have potential as a novel cell-free therapeutic option for MI.

Conventionally, most preclinical/clinical investigations on cell therapy for heart diseases have used single-dose delivery, mostly due to the technical difficulties of repeated administration and high lethality [74]. However, EVs lack some of the risks associated with cell-based therapies due to their low immunogenicity, minimal embolism risk, and biocompatibility. Furthermore, EVs can be delivered to the heart via various delivery routes, including intravenous, intracoronary and intramyocardial administration [75]. In the present study, we compared the therapeutic effects of HA-iMSC-EVs administered via various regime (i.e., delivery routes, dosages, and schedules). TTC/Evan’s blue staining showed that the infarct size was significantly reduced by HA-iMSC-EVs compared to that in the vehicle-treated animals, regardless of the delivery route. However, bioluminescence imaging revealed that most of the intravenously injected HA-iMSC-EVs were localized in off-target organs, including the brain, lungs, liver, and kidneys. Therefore, we decided to administer HA-iMSC-EVs via intramyocardial route to reduce the localization and possible side effects in non-cardiac tissues. We also tried to identify whether the repeated injection of HA-iMSC-EVs, considering clinically feasible platforms, could improve cardiac functions in comparison with single injection. However, we found that the repeated injection of HA-iMSC-EVs did not significantly improve systolic and diastolic cardiac functions. In histologic analysis, only capillary densities in the border zone and percent fibrosis of LV wall were significantly improved in the high (20 mg/kg) dose of repeated injection of HA-iMSC-EVs. Considering that the repeated injection of 10 mg/kg of HA-iMSC-EVs didn’t improve capillary densities and percent fibrosis compared with those of single injection of 20 mg/kg of HA-iMSC-EVs, we can infer that (1) the therapeutic concentration of 20 mg/kg in single injection group was set too higher than expected, and (2) the time point of secondary injection (at day 7 after the first one) may not be sufficient to reverse the inflammation and fibrosis, resulting in reduced dose-dependency in some functional parameters. Thus, we are planning to conduct further studies to investigate optimal applications such as HA-iMSC-EVs concentration and dose intervals, in order to enhance the HA-iMSC-EVs treatment regime.

## Conclusion

HA-iMSC-EVs improve cardiac repair via multiple cellular mechanisms including promoting capillary growth and attenuating tissue necrosis after MI. This strategy has potential to become an alternative option for cell-free therapeutics for cardiac repair.

### Supplementary Information

**Additional file 1: Fig S1.** Characterization of hiPSC-CM. a Characterization of hiPSC-CM was performed by flow cytometric analysis with cardiomyocyte markers including cardiac troponin T (cTnT), actinin alpha (α-actinin), alpha smooth muscle actin (α-SMA) and myosin light chain 2a (MLC2a). **Fig S2.** Protection effects of the HA-iMSC-EVs on primary neonatal rat cardiomyocyte. a-b EVs were treated to primary neonatal rat cardiomyocyte damaged with 500 μM of H2O2 for 2h. a Video were recorded for 15 seconds, and the beating area were marked yellow line. b After 48 h, relative viable cells were increased in EV-treated groups compared to PBS group. Mean ± SD, n= 3, **p < 0.01 vs PBS; ##p < 0.01 vs iMSC-EV. **Fig S3.** Heart rate during the cardiac function measurements. During cardiac ultrasound measurements, imaging was conducted on a temperature-controlled pad set at 40 degrees Celsius. Anesthesia was carefully administered using masks. Ultrasound system recorded M-mode images over a 3-second duration. Subsequently, heart rate calculations were derived from M-mode images captured at the 5-week time point. Mean ± SEM, n= 5.

## Data Availability

The materials can be provided upon request via email to the corresponding author.

## Abbreviations

AAR: Area at risk
ADSC: Adipose-derived stem cells
ATC: Anatomical therapeutic chemical
ATP: Adenosine triphosphate
CCK-8: Cell counting cell kit 8
cTnT: Cardiac troponin T
DAMP: Danger-associated molecular patterns
ECM: Extracellular matrix
EDPVR: End-diastolic pressure–volume relationship
EF: Ejection fraction
ESPVR: End-systolic pressure–volume relationship
EVs: Extracellular vesicles
FS: Fractional shortening
HA: Hyaluronic acid
HUVECs: Human umbilical venous endothelial cells
I/R: Ischemic reperfusion
IFN-γ: Interferon-gamma
IHD: Ischemic heart disease
IL-13: Interleukin 13
IL-1β: Interleukin-1 beta
IL-4: Interleukin 4
IM: Intramyocardial
iMSCs: Induced mesenchymal stem cells
iPSC-CM: Cardiomyocytes derived from iPSC
IV: Intravenously
LDH: Lactate dehydrogenase
LPS: Lipopolysaccharides
LV: Left ventricular
LVEDD: Left ventricular end diastolic diameter
LVEDV: Left ventricular end diastolic volume
LVEF: Left ventricular ejection fraction
LVESD: Left ventricular end systolic diameter
LVESV: Left ventricular end systolic volume
LVIDd: Left ventricular internal diastolic dimension
LVIDs: Left ventricular internal systolic dimension
MI: Myocardial infarction
MSCs: Mesenchymal stem cells
NLRP3: NACHT, LRR, and PYD domain-containing protein 3
NRCFs: Neonatal rat cardiac fibroblasts
NRCMs: Neonatal rat cardiomyocytes
NTA: Nanoparticle tracing analysis
PMA: Phorbol 12-myristate 13-acete
PV: Pressure–volume
ROS: Reactive oxidative species
SWT: Septal wall thickness
TEM: Transmission electronic microscopy
TGF-β: Transforming growth factor- beta
TTC: 2,3,5-Triphenyltetrazolium chloride
VEGF: Vascular endothelial growth factor
WJ: Wharton’s jelly
