# Supplementary material for: Hyaluronic acid stimulation of stem cells for cardiac repair: a cell-free strategy for myocardial infarct
Source: J Nanobiotechnology. 2024 Apr 4;22:149. doi: 10.1186/s12951-024-02410-x (PMC10993512; doi:10.1186/s12951-024-02410-x)
Supplement: Supplementary file 1 — Additional file 1: Fig S1. Characterization of hiPSC-CM. a Characterization of hiPSC-CM was performed by flow cytometric analysis with cardiomyocyte markers including cardiac troponin T (cTnT), actinin alpha (α-actinin), alpha smooth muscle actin (α-SMA) and myosin light chain 2a (MLC2a). Fig S2. Protection effects of the HA-iMSC-EVs on primary neonatal rat cardiomyocyte. a-b EVs were treated to primary neonatal rat cardiomyocyte damaged with 500 μM of H2O2 for 2h. a Video were recorded for 15 seconds, and the beating area were marked yellow line. b After 48 h, relative viable cells were increased in EV-treated groups compared to PBS group. Mean ± SD, n= 3, **p < 0.01 vs PBS; ##p < 0.01 vs iMSC-EV. Fig S3. Heart rate during the cardiac function measurements. During cardiac ultrasound measurements, imaging was conducted on a temperature-controlled pad set at 40 degrees Celsius. Anesthesia was carefully administered using masks. Ultrasound system recorded M-mode images over a 3-second duration. Subsequently, heart rate calculations were derived from M-mode images captured at the 5-week time point. Mean ± SEM, n= 5. [file 12951_2024_2410_MOESM1_ESM.docx]

**Supplementary Information**

Hyaluronic acid stimulation of stem cells for cardiac repair: a cell-free strategy for myocardial infarct

Seon-Yeong Jeong^1,^*, Bong-Woo Park^2^^,3^*, Jimin Kim^1^, Seulki Lee^1^, Haedeun You^1^, Joohyun Lee^1^, Susie Lee^2^, Jae-Hyun Park^2^, Jinju Kim^2^, Woosup Sim^2^, Kiwon Ban^4^, Joonghoon Park^5^, Hun-Jun Park^2,6,#^, Soo Kim^1,#^

^1^Brexogen Research Center, Brexogen Inc., Songpa‑gu, Seoul, 05855, South Korea.

^2^Department of Biomedicine & Health Sciences, The Catholic University of Korea.

^3^Catholic High-Performance Cell Therapy Center and Department of Medical Life Science, College of Medicine, The Catholic University of Korea, Seoul, 06591, Republic of Korea.

^4^Department of BiomedicalScience, City University of Hong Kong, Kowloon Tong, Hong Kong.

^5^Graduate School of International Agricultural Technology, Institutes of Green-Bio Science and Technology, Seoul National University, Pyeongchang, Gangwon-do, 25354, South Korea.

^6^Division of Cardiology, Department of Internal Medicine, Seoul St. Mary’s Hospital, The Catholic University of Korea.

*** These authors contributed equally**

**^#^ Address for Correspondence:**

Hun-Jun Park, MD., PhD., Seoul St. Mary’s Hospital, The Catholic University of Korea, 222 Banpo-daero, Seocho-gu, Seoul, 137-701, Republic of Korea. Email: cardioman@catholic.ac.kr

Soo Kim, PhD., Brexogen Research Center, Brexogen Inc., Songpa‑gu, Seoul 05855, South Korea. Email: sue.kim@brexogen.com**Supplementary Figures**


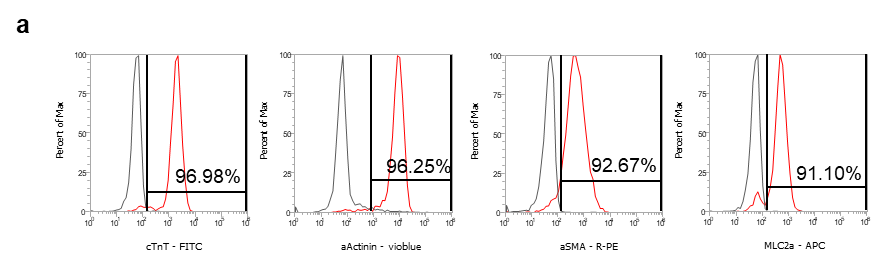


**Supplementary Fig. 1 Characterization of hiPSC-CM.**

**a** Characterization of hiPSC-CM was performed by flow cytometric analysis with cardiomyocyte markers including cardiac troponin T (cTnT), actinin alpha (α-actinin), alpha smooth muscle actin (α-SMA) and myosin light chain 2a (MLC2a).


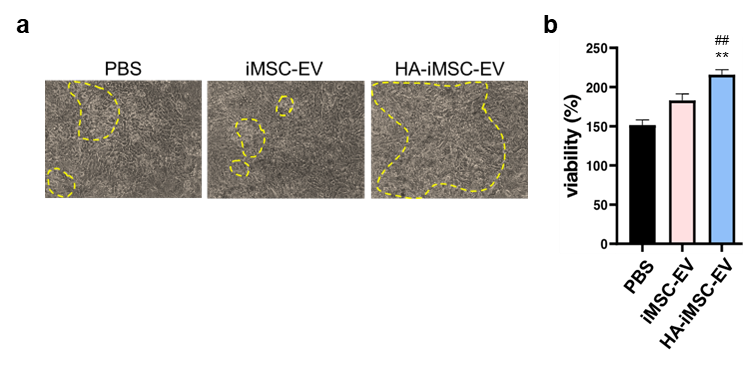


**Supplementary Fig. 2 Protection effects of the HA-iMSC-EVs on primary neonatal rat cardiomyocyte.**

**a-b** EVs were treated to primary neonatal rat cardiomyocyte damaged with 500 μM of H_2_O_2_ for 2h. **a** Video were recorded for 15 seconds, and the beating area were marked yellow line. **b** After 48 h, relative viable cells were increased in EV-treated groups compared to PBS group. Mean ± SD, n= 3, ^**^p < 0.01 vs PBS; ^##^p < 0.01 vs iMSC-EV.

**Supplementary Fig. 3 Heart rate during the cardiac function measurements.**

During cardiac ultrasound measurements, imaging was conducted on a temperature-controlled pad set at 40 degrees Celsius. Anesthesia was carefully administered using masks. Ultrasound system recorded M-mode images over a 3-second duration. Subsequently, heart rate calculations were derived from M-mode images captured at the 5-week time point. Mean ± SEM, n= 5.
